# Whole-Genome Sequences of Seven Strains of *Bacillus cereus* Isolated from Foodstuff or Poisoning Incidents

**DOI:** 10.1128/genomeA.00435-16

**Published:** 2016-06-09

**Authors:** Julien Crovadore, Gautier Calmin, Jenna Tonacini, Romain Chablais, Bruno Schnyder, Ute Messelhäußer, François Lefort

**Affiliations:** a Plants and Pathogens Group, Research Institute Land Nature and Environment, hepia, HES-SO University of Applied Sciences and Arts Western Switzerland, Jussy, Geneva, Switzerland; b Faculty of Engineering and Architecture, HES-SO University of Applied Sciences and Arts Western Switzerland, Delémont, Switzerland; c Institute of Life Technologies, hevs, HES-SO University of Applied Sciences and Arts Western Switzerland, Sion, Switzerland; d Bavarian Health and Food Safety Authority, Laboratory of Food Microbiology, Oberschleißheim, Germany

## Abstract

We present here the whole shotgun genome sequences of seven strains of *Bacillus cereus* isolated from foodstuff samples or food poisoning incidents.

## GENOME ANNOUNCEMENT

*Bacillus cereus* Frankland and Frankland 1887 is a Gram-positive, rod-shaped, spore-forming, and motile facultative aerobic bacterium. This soil inhabitant is an opportunistic human pathogen that causes food poisoning and belongs to the *Bacillus cereus* senso lato group, along with *Bacillus anthracis*, *Bacillus thuringiensis*, and other species of medical, industrial, or agricultural interests (1, 2). Due to the thermal resistance of endospores, this bacterium may cause severe nausea, vomiting, and diarrhea in infected individuals. The emetic syndrome is caused by cereulide (3, 4), and the diarrheal syndrome is due to three toxins: hemolysin BL (Hbl), nonhemolytic enterotoxin (Nhe), and cytotoxin K (CytK) (5). Since the first genomes were published (6, 7), 247 genomes are now available in the NCBI genome database.

Genomic DNAs of these 7 *Bacillus cereus* strains were extracted from pure cultures following an adapted protocol (8). Libraries were performed using the Nextera XT kit (Illumina, USA). All genomes were sequenced within one Illumina MiSeq run at 2- × 250-bp read length. One genome was resequenced with the MinION MKI nanosequencer (Oxford Nanopore Technologies, United Kingdom) (9). Sequencing data analyzed with the Metrichor Agent (v2.37) were assembled with Illumina reads of these genomes. Trimming and quality control were performed with FastQC (10). Genome assemblies were computed with SPAdes Genome assembler 3.6.2 (11). Resulting contigs arranged with BioEdit (12) were analyzed with QUAST (13). Genomes were screened for plasmids with PlasmidFinder (14). Automated gene annotation was carried out by the NCBI Prokaryotic Genome Automatic Annotation Pipeline PGAAP (15) and reviewed with RAST version 2.0 (16).

The genome coverage varied between 83-fold (MB-1) and 137-fold (DSM2302) of genome length. Total genome lengths ranged between 5,393,440 bp (DSM2302) and 5,737,072 bp (MB-8-1), within the known diversity. GC content ranged from 35.14% (MB-1) to 35.38% (MB-21). While it was usually thought that toxic phenotypes relied on plasmids (3), no strain harbored a plasmid. Five strains contained a complete cereulide synthetase operon with *cesa* and *cesb* genes, and DSM2302 and MB-1 lacked this operon. This was confirmed by a biochemical assay of cereulide and matrix-assisted laser desorption ionization–time of flight (MALDI-TOF) detection. All strains harbored antibiotic resistance genes such as *SatA* for streptothricin; *EF-G* and *Tet-like* for tetracycline; *FosB* for fosfomycin; *BL*, *BLI* and *BLA* for beta-lactamase; *parC*, *pare*, *gyrA*, and *gyrB* for fluoroquinolones (with DSM2302 having an additional *Lde* gene). The vancomycin resistance gene *vanW* was found in all strains. An almost complete *Listeria* pathogenicity island LIPI-1 with 4 genes (*PlcA*, *LLO*, *MpL*, and *PlcB*) was retrieved in all strains except the strain DSM2302, which lacked the *LLO* gene. Finally, all strains were equipped with genes of the nonhemolytic enterotoxin A, enterotoxin C, and 3 to 4 to genes encoding not yet described enterotoxins. The strain MB-18 had an additional gene for the cytotoxin K enterotoxin. The sequences of these seven genomes will add to the understanding of the pathogenicity of this species.

### Nucleotide sequence accession numbers.

All genome sequences have been deposited at GenBank under the accession numbers reported in Table 1.

**TABLE 1:** Nucleotide sequence accession numbers

Strain name	GenBank accession no.	Contig accession no.
DSM2302	LQZO00000000	LQZO01000001–LQZO01000278
MB.1	LQZN00000000	LQZN01000001–LQZN01000194
MB.15	LQZM00000000	LQZM01000001–LQZM01000266
MB.17	LQZL00000000	LQZL01000001–LQZL01000200
MB.18	LRAA00000000	LRAA01000001–LRAA01000328
MB.8-1^a^	LQZX00000000	LQZX01000001–LQZX01000354
MB.21	LQZW00000000	LQZW01000001–LQZW01000359

^a^ This genome resequenced with the MinION MKI.
